# Unilateral Optic Neuropathy and Acute Angle-Closure Glaucoma following Snake Envenomation

**DOI:** 10.1155/2015/687829

**Published:** 2015-01-29

**Authors:** Osman Okan Olcaysu, Kenan Cadirci, Ahmet Altun, Afak Durur Karakaya, Huseyin Bayramlar

**Affiliations:** ^1^Clinic of Ophthalmology, Erzurum Education and Research Hospital, 25240 Erzurum, Turkey; ^2^Clinic of Internal Medicine, Erzurum Education and Research Hospital, 25240 Erzurum, Turkey; ^3^Clinic of Ophthalmology, Fatih Sultan Mehmet Education and Research Hospital, 34752 Istanbul, Turkey; ^4^Clinic of Radiology, Erzurum Education and Research Hospital, 25240 Erzurum, Turkey; ^5^Department of Ophthalmology, Medical Faculty, Medeniyet University, 34730 Istanbul, Turkey

## Abstract

*Purpose*. We aimed to describe a unique case in which a patient developed unilateral optic neuritis and angle-closure glaucoma as a result of snake envenomation. *Case Report*. Approximately 18 hours after envenomation, a 67-year-old female patient described visual impairment and severe pain in her left eye (LE). The patient's best corrected visual acuity was 10/10 in the RE and hand motion in the LE. Cranial magnetic resonance imaging showed signs of neuropathy in the left optic nerve. In the LE, corneal haziness, closure of the iridocorneal angle, and mild mydriasis were observed and pupillary light reflex was absent. Intraocular pressure was 25 mmHg and 57 mmHg in the RE and LE, respectively. The patient was diagnosed with acute angle-closure glaucoma in the LE. Optic neuropathy was treated with intravenous pulse methylprednisolone. Left intraocular pressure was within normal range starting on the fourth day. One month after the incident, there was no sign of optic neuropathy; relative afferent pupillary defect and optic nerve swelling disappeared. *Conclusions*. Patients with severe headache and visual loss after snake envenomation must be carefully examined for possible optic neuropathy and angle-closure glaucoma. Early diagnosis and treatment of these cases are necessary to prevent permanent damage to optic nerves.

## 1. Introduction

According to the World Health Organization, 2.5 million cases of snake envenomation occur each year, 125,000 of which are fatal [1]. Venomous snakebites are more common in tropical rural areas. It is estimated that there are around 2,500 to 3,000 venomous snake species in the world, a third of which are believed to possess lethal venom. Most cases of poisoning happen during summer [2]. Envenomation can lead to neurological or hematologic dysfunction. Neurotoxic snake venom especially affects neuromuscular junctions in the peripheral nervous system; cranial nerves may also be affected. Ptosis and external ophthalmoplegia may occur. Accommodation paralysis, optic neuropathy, ocular necrosis, keratomalacia, uveitis, and vision loss due to cortical infarction have also been reported, although they are less common complications [3, 4]. Subconjunctival hemorrhage, hyphema, vitreous hemorrhage, and retinal hemorrhage due to hematologic dysfunction have also been reported as a result of hemotoxic snake venom [3, 5]. Here we present a case that developed unilateral optic neuropathy and acute angle-closure glaucoma following snake envenomation.

## 2. Materials and Methods

A 67-year-old female was bitten by a snake on the left ankle while working in an agricultural field. According to information provided by the patient and witnesses, the snake was identified as* Vipera lebetina*. The patient began to lose consciousness four hours after the snakebite. She was admitted to the local hospital and given snake antivenom and emergency medical supportive care and then was urgently transferred to the Erzurum Training and Research Hospital intensive care unit.

**Figure 1:**
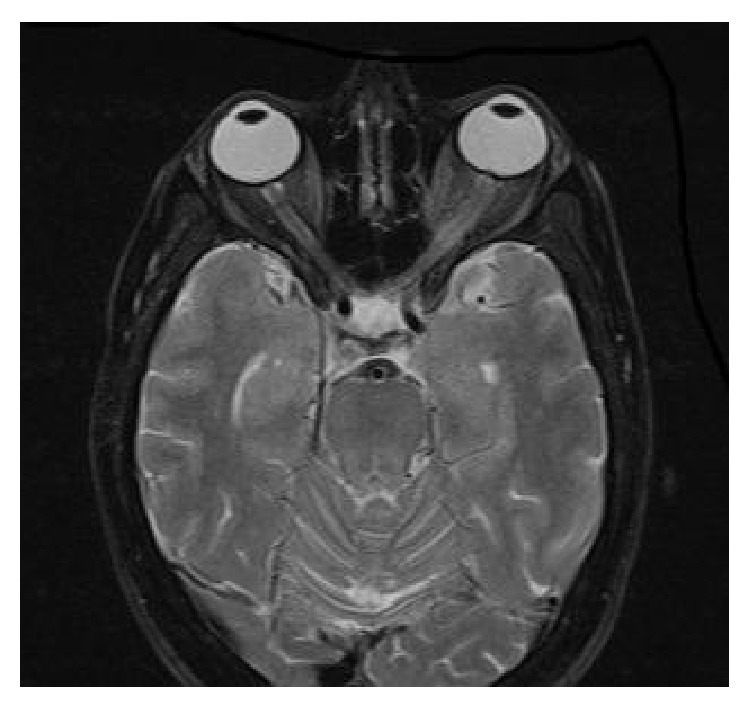
Cranial magnetic resonance imaging of neuropathy in the left optic nerve.

When admitted to our clinic, the patient felt inclined to sleep. The patient's Glasgow Coma score was 12, brachial pulse was 130 bpm, systemic blood pressure was 100/60 mmHg, and breathing rate was within normal range. Fang marks were visible on the patient's ankle and there were signs of bleeding and edema. Blood analysis showed normal hemoglobin levels (14 g/dL), normal platelet count (140,000/*μ*L), lowered fibrinogen levels (143 mg/dL; normal range: 248–328 mg/dL), and prolonged prothrombin time (>200 seconds; normal range: 25–40 seconds). Kidney function analysis, abdominal ultrasonography, and electrocardiography (ECG) results were normal. Cranial magnetic resonance imaging (MRI) was normal with the exception of signs of neuropathy in the left optic nerve (Figure 1).

**Figure 2:**
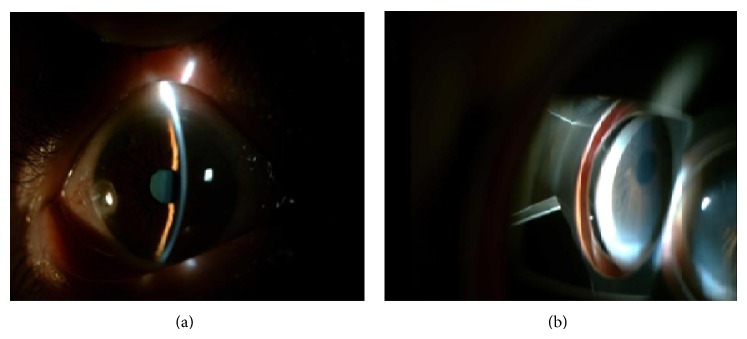
Ciliar injection in the conjunctiva (a), closure of iridocorneal angle (b), and narrow anterior chamber (a) in the left eye.

**Figure 3:**
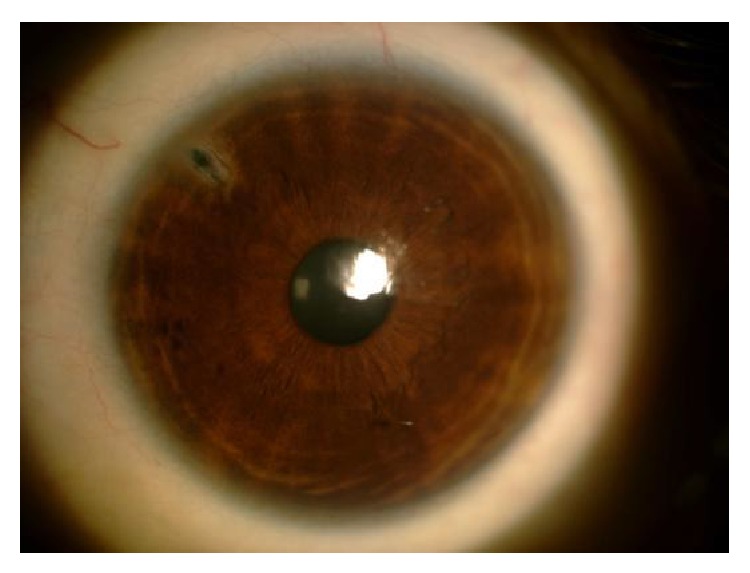
YAG-laser peripheral iridotomy on the left eye.

Approximately 18 hours after envenomation, patient gained consciousness and described severe pain and lowered visual acuity in the LE. In the ophthamalogic examination, the patient had best corrected visual acuity (BCVA) of 10/10 (assessed by Snellen chart) in the right eye (RE) and hand motion in the LE. In the anterior segment of the RE, the iridocorneal angle was narrow and the posterior segment showed no abnormality. In the LE, ciliar injection in the conjunctiva, corneal haziness, closure of iridocorneal angle, narrow anterior chamber, and mild mydriasis were observed and pupillary light reflex was absent (Figures 2(a) and 2(b)). Although it was not possible to examine the posterior segment, possibly due to corneal edema, fundus reflex was present. Intraocular pressure was measured using Goldmann applanation tonometer and was 25 mmHg and 57 mmHg in the RE and LE, respectively. The patient was diagnosed with acute angle-closure glaucoma in the LE. The patient had no history of the use of medications with anticholinergic effect (antidepressant, antipsychotic, antihistaminic, or anti-Parkinsonian agents) or atropine or similar drugs used in general anesthesia. The patient received topical 2% pilocarpine hydrocloride (Pilosed, Bilim Medicine, Istanbul, Turkey) four times a day; topical dorzolamide hydrocloride-timolol maleate solution (Cosopt, Merck, USA) twice a day; topical prednisolone acetate (Predforte, Allergan, CA, USA) five times a day; intravenous 150 cc of 20% mannitol at a dose of 1-2 mg/kg; and 250 mg acetazolamide (Diazomid, Sanofi Aventis, Istanbul, Turkey) tablet taken orally twice a day. Intravenous 1 gr/kg pulse methylprednisolone (Prednol -L 250 mg, Mustafa Nevzat, Gayrettepe, Istanbul, Turkey) was administered to treat optic neuropathy. YAG-laser peripheral iridotomy was performed on the LE after partial resolution of the corneal edema (Figure 3).

**Figure 4:**
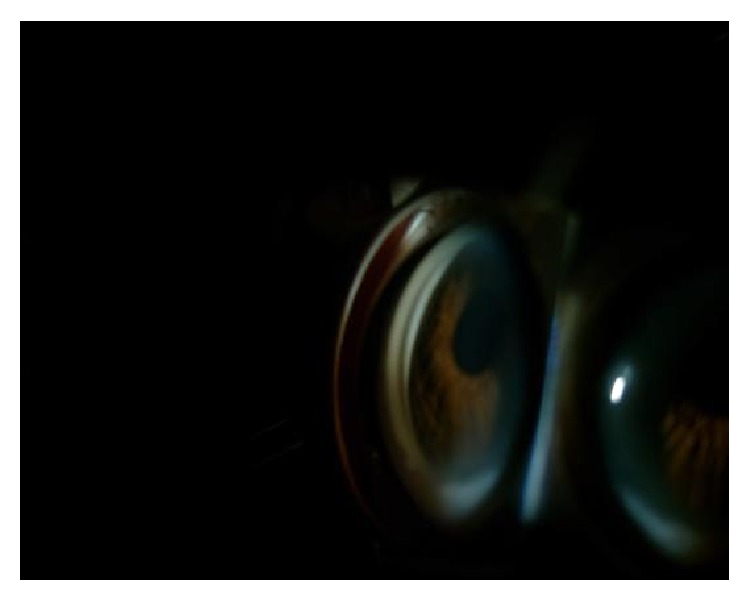
The iridocorneal angle is Grade 1 according to Shaffer classification.

On the fourth day, patient's BCVA was 10/10 in the RE and 0.05 in the LE. The anterior and posterior segments of the RE continued to show no abnormalities. In the LE, the cornea was clear and there was slight pupil dilation. The iridocorneal angle was measured as Grade 1 according to Shaffer classification (Figure 4). In the posterior segment of the LE, the optic disc appeared normal. Intraocular pressure was 19 mmHg and 10 mmHg in the RE and LE, respectively. Oral acetazolamide, topical prednisolone acetate, and intravenous pulse methylprednisolone were discontinued and the patient received 1 mg/kg/day oral prednizolone for two weeks (Prednol 16 mg, Mustafa Nevzat, Gayrettepe, Istanbul, Turkey).

**Figure 5:**
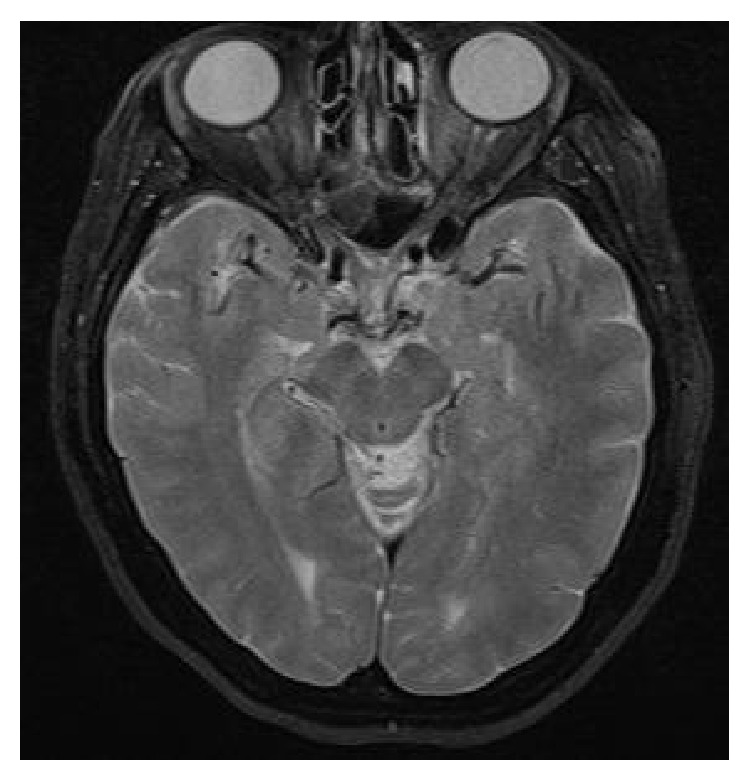
Cranial magnetic resonance imaging one month later (the neuropathy in the left optic nerve was regressed).

After two weeks, the BCVA was 10/10 and 4/10 and intraocular pressure was 12 mmHg and 8 mmHg in the RE and LE, respectively. With the exception of mild mydriasis in the LE, the anterior and posterior segments of both eyes appeared normal. At one month, BCVA in the LE had improved to 5/10. Intraocular pressure was 12 mmHg and 8 mmHg in the RE and LE, respectively. Furthermore, cranial MRI conducted one month later showed marked regression of the optic neuropathy in the LE (Figure 5).

## 3. Discussion

Viperine snake venom is a complex mixture including hemotoxins, which especially initiate the coagulation cascade and neurotoxins that act on synapses [3, 6]. Optic neuropathy is a rare complication of snake envenomation and, when it occurs, it is almost always bilateral, although it may present unilaterally [6–9].

There are only two case reports in the literature of glaucoma caused by a venomous snakebite. One of the reports describes bilateral angle-closure glaucoma, whereas the other describes unilateral ghost cell glaucoma [10, 11]. In the case of bilateral angle-closure glaucoma, the hemorrhagic aspect was more pronounced. The Viperidae venom caused serious vasospasm, endothelial damage, and increased vascular permeability and resulted in edema in the ciliary body and iris. Due to the ciliary body and iris edema, aqueous humour drainage to the anterior chamber was blocked, resulting in bilateral secondary angle-closure glaucoma [10]. In the other case, the snakebite caused vitreous hemorrhage, resulting in blood and blood components to collect in the anterior chamber, thereby increasing intraocular pressure. Intraocular pressure could not be lowered to normal range with topical antiglaucoma treatments, necessitating a posterior vitrectomy to normalize the patient's intraocular pressure [11].

Here, we report a unique case of unilateral optic neuropathy and unilateral angle-closure glaucoma due to snake envenomation. Ghost cell glaucoma was ruled out, as there was no sign of intraocular hemorrhage. Furthermore, there was no sign of inflammation or posterior synechiae, which eliminated iridocyclitis. When scrutinizing possible cause of unilateral angle-closure glaucoma, the following were considered: (1) although both eyes showed narrow asymmetric anterior chamber angles, the angle was markedly narrower in the LE; (2) the hemotoxic properties of Viperidae venom cause vascular damage and increase vascular permeability, which can lead to the formation of edema in the ciliary body and iris, causing the lens-iris diaphragm to move forward and block the pupil; (3) as the patient's eyes were predisposed to angle closure, the sympathetic reaction caused by the venom or the snakebite itself may have induced mydriasis, which in turn caused pupillary block and led to iridocorneal angle closure.

To the best of our knowledge, this case report is the first instance described in literature of a patient developing unilateral angle-closure glaucoma in response to snake envenomation. This case highlights the need to carefully follow patients with severe headache and visual impairment after snake envenomation, as the possibility of optic neuropathy and angle-closure glaucoma exists.

## 4. Conclusions

Early diagnosis and treatment of these cases are necessary to prevent permanent damage to optic nerves.
